# Prognosis of adrenalectomy guided by computed tomography versus adrenal vein sampling in patients with primary aldosteronism: A systematic review and meta‐analysis

**DOI:** 10.1111/jch.14395

**Published:** 2022-01-22

**Authors:** Yi Yan, Hui‐Wen Sun, Yue Qi

**Affiliations:** ^1^ Beijing An Zhen Hospital Capital Medical University Beijing China; ^2^ Beijing Institute of Heart Lung and Blood Vessel Diseases Beijing China

**Keywords:** adrenal vein sampling, adrenalectomy, computed tomography, primary aldosteronism, prognosis

## Abstract

Adrenal vein sampling (AVS) is recommended to be the gold standard for patients with unilateral subtypes of primary aldosteronism to clinical diagnosis and surgery therapy. However, it is uncertain whether AVS is better for prognosis than computed tomography (CT), which is the most widely used. Pubmed, Embase, and Cochrane Library were searched for articles with no start date restriction. The last search was conducted on Jun 15, 2021. Eligible studies compared the distinct subtypes of primary aldosteronism by AVS with CT (as a control group) and reported the prognosis at follow‐up. Evaluation of cohort studies referred to Newcastle ‐ Ottawa Quality Assessment Scale, and randomized controlled trials referred to Updated Cochrane Collaboration tool. A random‐effect model or fixed‐effect model was chosen according to the heterogeneity test. All processes were performed following the PRISMA 2020 statement. Eleven studies were identified, including 1325 patients based on AVS and 907 patients based on CT. Compared with patients guided by CT, patients who underwent AVS had an increased possibility of complete biochemical success (odds ratio [OR] 2.78, 95% CI 1.88–4.12) and a decreased chance of absent biochemical success (OR 0.23, 95% CI 0.13–0.40) at follow‐up. Nevertheless, the rate of complete clinical success (OR 1.09, 95% CI 0.89–1.35) and absent clinical success (OR 0.96, 95% CI 0.68–1.33) had no significant difference. Therefore, distinguishing subtypes by AVS for early treatment may be crucial since it can promote biochemical improvement.

## 1 INTRODUCTION

Primary aldosteronism is one of the most important secondary hypertension causes, approximately consisting of 3.2–12.7% general hypertensive patients^1^ and 3.0–13.8% normotension population.^2^, ^3^, ^4^ Compared with essential hypertension, patients with primary aldosteronism suffer more severe damage of cardio‐cerebrovascular and renal vessel,^5^, ^6^ leading to a higher risk of coronary artery disease, stroke, atrial fibrillation, heart failure, and kidney disease, due to the direct effect of aldosterone on the cardiovascular system, including arterial wall inflammation, remodeling, and fibrosis.^7^ A growing body of evidence implies that accurate diagnosis and treatment can reverse target organ damage.^8^ To choose optimal clinical management, correctly dividing primary aldosteronism into unilateral or bilateral subtypes is the key factor.^9^ Surgery is indicated for unilateral subtypes, and lifelong use of mineralocorticoid antagonists is suitable for patients with bilateral subtypes.^9^ There are two main methods to distinguish the subtypes of primary aldosteronism, adrenal vein sampling (AVS) and computed tomography (CT).

CT is widely used to distinguish unilateral from bilateral primary aldosteronism in clinical practice, yet it can be misleading. A systematic review asserted that 14.6% of patients are diagnosed as unilateral by CT while AVS diagnose as bilateral.^10^ The adenoma showed by CT on the contrary side occupies 3.9%, which will lead to the wrong removal of the contralateral adrenal.^10^ AVS is an invasive way to detect both adrenal glands' secretory function,^11^ which is considered the gold standard for determining the laterality of primary aldosteronism. Endocrine Society guidelines recommend that patients with primary aldosteronism should undergo AVS unless meeting the following conditions at the same time: age < 35 years old, spontaneous hypokalemia, significantly increased aldosterone concentration, and imaging showing adrenal cortical adenomas.^9^ However, some current evidence has cast uncertainty of the benefits after adrenalectomy by AVS.^12^ In 2016, a randomized diagnostic trial in 92 patients (46 used CT and 46 used AVS) found no significant difference in blood pressure control and biochemical improvement at 1‐year follow‐up.^13^ But recently, two international multicenter cohort studies showed patients after AVS classification^14^ could obtain a better prognosis, reflected in a higher rate of cure of hypertension and a decreased likelihood of achieving complete biochemical success.^15^ Therefore, the results of the prognosis of adrenalectomy guided by CT versus AVS in patients with primary aldosteronism are highly heterogeneous. All single‐center studies have fewer than 50 patients in at least one group.

Given the high morbidity of primary aldosteronism and the life‐long severe consequences caused by the wrong resection, it is necessary to conduct a systematic review and meta‐analysis including all published cohort studies and randomized controlled trials to explore clinical and biochemical benefits after adrenalectomy among patients with unilateral primary aldosteronism classified under CT and AVS guidance.

## 2 METHODS

### 2.1 Identification and selection criteria

Pubmed, Embase, and Cochrane Library were searched for relevant articles using terms associated with primary aldosteronism and adrenalectomy (eg, “hyperaldosteronism” “primary aldosteronism” or “Conn syndrome”, and the following terms: “surgery” or “adrenalectomy”) from inception to January 21, 2021. A traceability search on the references of reviews and related documents of each included study was conducted. The last search was conducted on June 15, 2021, and a new study was discovered, but it was a duplicate report, so no new studies were included.^16^ The author Y.Y. and H.W.S. conducted the searches independently. If there is any inconsistency, Y.Q. was responsible for the resolution.

A study was eligible for inclusion if (1) patients diagnosed as primary aldosteronism. The diagnostic criteria for primary aldosteronism were described in detail in Tables S1; (2) unilateral adrenalectomy was recognized by CT or AVS; and (3) reported results of biochemical or clinical outcome at follow‐up. A study was excluded if (1) distinguish the subtypes of primary aldosteronism by PET/CT; (2) unreported data on sample size or standard deviation; and (3) duplicated reports.

### 2.2 Data extraction and quality assessment

Two unmasked independent reviewers (Y.Y. and H.W.S.) extracted the study data and assessed the bias risk. Data extraction including the following six aspects: article information (authors, year of publication, and country); study information (study type, years of data, sample size, and whether selective grouping); baseline features (number of antihypertensive drugs, duration of hypertension); duration of follow‐up; the diagnostic and classification criteria of primary aldosteronism (CT parameters, AVS operation details, cut‐off values); the definition of clinical and biochemical success (Table S5). Evaluation of cohort studies referred to Newcastle ‐ Ottawa Quality Assessment Scale.^17^ The Updated Cochrane Collaboration tool was used to assess the eligible randomized controlled trial's methodological quality (Table S2).^18^ Any disagreements in abstracted data and assessment quality were adjudicated by a third reviewer (Y.Q.).

### 2.3 Outcomes

The outcomes for the analysis included the rate of complete, partial, or absent success of clinical and biochemical indicators, as well as the value of clinical and biochemical indicators. Clinical indicators during follow‐up included blood pressure, the number of antihypertensive drugs. Biochemical indicators included serum potassium, plasma aldosterone concentration, plasma renin activity, and aldosterone‐to‐renin ratio.

Williams and coworkers used the Delphi method to establish the Primary Aldosteronism Surgical Outcome (PASO) international consensus.^19^ According to PASO, complete clinical success is normal blood pressure lacking antihypertensive medication. Absent clinical success suggests unchanged or increased blood pressure with either the same or increasing amount in antihypertensive medicines. Complete biochemical success indicates a correction of hypokalemia, firstly. And the other condition, normalization of the aldosterone‐to‐renin ratio, could be replaced, in patients with a raised aldosterone‐to‐renin ratio post‐surgery, by suppressed aldosterone secretion in a confirmatory test. Absent biochemical success refers to persistent hypokalemia or persistent increased aldosterone‐to‐renin ratio, or both, as well as failure to suppress aldosterone secretion in the post‐surgery confirmatory test. Besides, partial success lies between complete and absent. The definitions of clinical and biochemical success in this study were summarized in Table S3.

### 2.4 Data analysis and synthesis

Categorical variables are reported as n/N (%). Continuous variables were expressed as mean (SD) or median (IQR). For the data that cannot be directly included in the meta‐analysis, data conversion was carried out according to Luo and coworkers^20^ and Wan and coworkers.^21^ Mantel‐Haenszel method for categorical variables and Inverse‐Variance method for continuous variables calculated the odds ratio with 95% confidence interval (95% CI). Heterogeneity was assessed. When I^2^ ≥ 75%, a random‐effect model was applied; otherwise, a fixed‐effect model was used. Subgroup analysis was conducted according to whether AVS is performed with guidance by CT results, and the detailed criteria were shown in Table S4. Sensitivity analysis was conducted using a random‐effect model, excluding the studies with the largest sample size, high‐risk bias studies, and the randomized controlled trial. Funnel plots were used to check for research publication bias.

The systematic review and meta‐analysis was performed following the PRISMA 2020 statement.^22^ All analyses did with Revman 5.3. *p* < 0.05 was considered statistically different, based on the two‐tailed test.

### 2.5 Role of the funding source

The funder of the study had no role in study design, data collection, data analysis, data interpretation, or writing of the report. The corresponding author Y.Q. had full access to all the data in the study and had final responsibility for the decision to submit for publication.

## 3 RESULTS

### 3.1 Study characteristics

**FIGURE 1:**
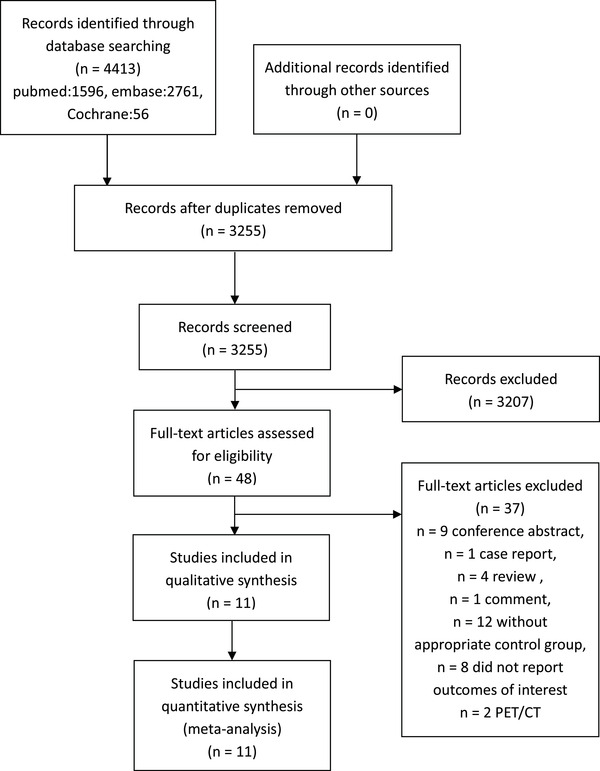
Flow chart of literature search and study selection. The diagram summarizes search results from inception to January 21, 2021. The search was updated on June 15, 2021. Pubmed new found 47 records, Embase 75 records, and Cochrane library 1 records. All 123 records were screened for eligibility, but no new studies meeting the inclusion criteria were identified

Eleven studies^13^, ^14^, ^15^, ^23^, ^24^, ^25^, ^26^, ^27^, ^28^, ^29^, ^30^ were included in the analysis (detailed literature search described in Figure 1), including 1325 primary aldosteronism patients who underwent adrenalectomy guided by AVS and 907 patients guided by CT. Characteristics of the studies meeting the criteria and included in the final meta‐analysis were in Tables 1 and S5. There were one randomized controlled trial and ten cohort studies. Two cohort studies were at high risk of bias, and others were low or moderate risks (Table S2). Six of the seven items in the Updated Cochrane Collaboration tool for the randomized controlled trial were low risks, so the overall evaluation was low.

**TABLE 1:** Characteristics of the studies meeting the inclusion criteria

	Country	Study type	Data	Selective grouping	Sample size (CT vs. AVS)	Duration of hypertension (CT vs. AVS; years)	Antihypertensive drugs in diagnosis (CT vs. AVS)	Duration of follow‐up (months)
Dekkers and coworkers (2016)^13^	Dutch, Polish	Randomized controlled trial	2010‐2013	No	46 vs 46	NA	NA	>12
Ma and coworkers (2020)^23^	China	Cohort	2015‐2018	No	195 vs 40	2 vs 5	No. 1.4 vs 2	3‐48
Nwariaku and coworkers (2006)^24^	USA	Cohort	2000‐2004	No	7 vs 25	NA	NA	NA
Pirvu and coworkers (2014)^25^	France	Cohort	1998‐2012	Yes	53 vs 9	NA	NA	3‐88
Rossi and coworkers (2019)^14^	Italy, France, Germany, Czech Republic, Japan, Netherlands, Canada, Australia, Russia, Spain, China, UK	Cohort	2005‐2015	No	151 vs 492	NA	NA	6‐12
Tan and coworkers (2006)^26^	USA	Cohort	1995‐2004	Yes	54 vs 11	NA	NA	0.5‐1
Thiesmeyer and coworkers (2020)^27^	USA, France	Cohort	2004‐2019	No	80 vs 45	5 vs 8	No. 3 vs 2	5.5
Williams and coworkers (2018)^15^	Slovenia, Poland, Germany, Australia, Japan, Italy	Cohort	1994‐2016	No	235 vs 526	NA	DDD 2.7 vs 2.7	6‐12
Yeung and coworkers (2020)^28^	USA	Cohort	2005‐2015	Yes	26 vs 6	NA	No. 2.76 vs 2.74	43
Zarnegar and coworkers (2007)^29^	USA	Cohort	1996‐2005	Yes	30 vs 29	12.3 vs 11	No. 3 vs 2.8	6
Zhu and coworkers (2016)^30^	China	Cohort	2005‐2014	Yes	30 vs 96	4 vs 6	No. 2 vs 3	37

The sample size was only included in patients undergoing adrenalectomy under CT or AVS guidance and was not the study's total sample size.

*Abbreviations*: DDD, Defined daily dose; NA, not available; No., the number of antihypertensive drugs.

### 3.2 Clinical outcomes

**FIGURE 2:**
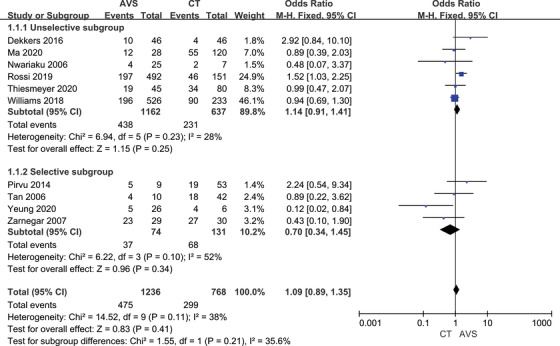
Complete clinical success rate with CT versus AVS guided

The rate of clinical success was shown in Table 2. The complete clinical success rate was 38.9% by CT‐guided and 38.4% by AVS‐guided based on ten studies. The partial clinical success rate was 47.8% and 50.2% summarized eight studies. In nine studies, the absent clinical rate was 11.0% and 9.6%. Patients guided by AVS had lower systolic blood pressure (‐2.33, 95% CI ‐3.88 to ‐0.77; Figure S1), while other clinical outcomes were no significant difference. The rate of complete clinical success (OR 1.09, 95% CI 0.89–1.35; Figure 2), partial clinical success (OR 0.94, 95% CI 0.76–1.16; Figure S2), absent clinical success (OR 0.96, 95% CI 0.68–1.33) had no benefits from AVS. Diastolic blood pressure (‐0.78, 95% CI ‐1.74 to 0.18) and the number of antihypertensive drugs (0.10, ‐0.10 to 0.29) also had no significant difference. However, in the selective subgroup, AVS can reduce the rate of absent clinical success (OR 0.37, 95% CI 0.15–0.95). There was no significant difference in complete clinical success in each subgroup.

**TABLE 2:** The clinical and biochemical success rate based on CT or AVS

	CT	AVS
Clinical success		
Complete	38.9%	38.4%
Partial	47.8%	50.2%
Absent	11.0%	9.60%
Biochemical success		
Complete	79.4%	91.2%
Partial	9.68%	6.67%
Absent	9.59%	1.93%

The sum of the success rates was not equal to 100% because some studies did not report all the success rates, or the data provided by them were not suitable for classification.

### 3.3 Biochemical outcomes

**FIGURE 3:**
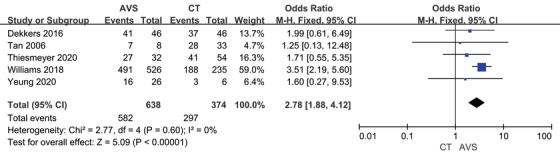
Complete biochemical success rate with CT versus AVS guided

**FIGURE 4:**
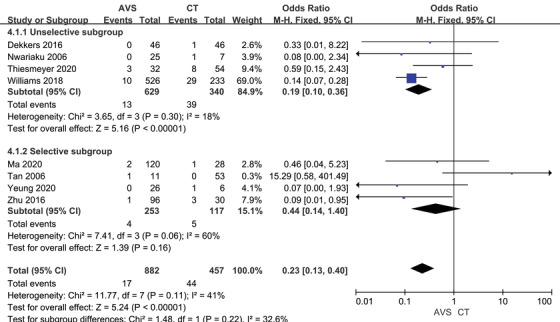
Absent biochemical success rate with CT versus AVS guided

The complete biochemical success rate was 79.4% by CT‐guided and 91.2% by AVS‐guided, the partial biochemical success rate was 9.68% and 6.67%, and the absent biochemical success rate was 9.59% and 1.93% (Table 2). Before adrenalectomy, patients who underwent AVS showed an increased possibility of complete biochemical success (OR 2.78, 95% CI 1.88–4.12; Figure 3) when analyzing five reports with 1010 patients. Similarly, AVS‐guided patients had a decreased likelihood of absent biochemical success after gathered eight reports with 1339 patients (OR 0.23, 95% CI 0.13–0.40; Figure 4), especially in the unselective subgroup. Patients guided by AVS got a lower plasma aldosterone concentration summarized from four studies(‐0.91, 95% CI ‐1.75 to ‐0.07). However, the AVS‐guided did not affect the partial biochemical success (OR 0.63, 95% CI 0.38–1.05, Figure S2) and serum potassium (‐0.04, 95% CI ‐0.34 to 0.25, Figure S1).

Only one study reported the aldosterone‐renin ratio at follow‐up and found that patients guided by AVS had a significantly lower ratio compared with patients guided by CT. Among three studies that reported the plasma renin activity, one large‐sample study reported AVS‐guided patients had higher activity than CT‐guided patients, while the other two small‐sample studies found no difference (Table S6).

### 3.4 Sensitivity analyses

Sensitivity analysis was conducted using a random‐effects model for clinical and biochemical success rates, and there were no statistical changes in the six indicators (Figure S3). After excluding the study with the largest sample size, a higher rate of complete biochemical success (OR 1.74, 95% CI 0.85–3.53) was found in 112 patients guided by AVS than 139 patients with CT‐guided, despite borderline significance. Further excluding the randomized controlled trial by Dekkers and coworkers, the AVS‐guided group still had a significant advantage (OR 2.91, 95% Cl 1.91–4.41).

Meanwhile, after excluding the largest sample study, high‐risk bias studies, or the randomized controlled trial, there was still a significantly lower rate of absent biochemical success in patients with AVS than those with CT guidance (OR 0.43, 95% CI 0.19–0.99; OR 0.19, 95% CI 0.10–0.34; OR 0.22, 95% CI 0.13–0.39) (Figure S3).

### 3.5 Reporting biases

No obvious bias was found for the four main indicators: complete clinical success, absent clinical success, complete biochemical success, and absent biochemical success (Figure S4).

## 4 DISCUSSION

Discordant findings exist of an effect between the CT scanner and AVS on both biochemical and clinical cure after adrenalectomy for primary aldosteronism. A systematic review of the literature provided insight into this process. This study was based on 11 studies with 1325 primary aldosteronism patients who underwent adrenalectomy guided by AVS and 907 patients guided by CT scanner, and confirmed for the first time in a large meta‐analysis that AVS improves the biochemical aspect of the syndrome but does not predict improvement in the clinical picture after surgery, which contributes to resolving inconsistent findings of clinical studies by providing higher‐level evidence. The other unique contribution is providing CT‐guided remission rates for adrenalectomy in a large sample from this systematic review and meta‐analysis. Based on an international consensus on outcome measures, only studies guided by AVS have been published so far. This systematic review and meta‐analysis obtained similar remission rates to those guided by AVS in PASO study, which indirectly demonstrates the reliability of the CT‐guided clinical remission rate.

AVS can directly detect the bilateral adrenal glands' function and is defined as the gold standard by the guidelines. Therefore, if treatment is given based on the correct diagnosis, better benefits should be obtained. Our results also suggest that it has a clear benefit for biochemistry, but we did not find its clinical benefit. There are two aspects of possible reasons.

On the one hand, the technique and interpretation criteria of AVS are challenging.^31^ The absence of ACTH stimulation will increase intubation failure,^32^ and using ACTH stimulation will increase the misdiagnosis rate of AVS.^33^ Different judgment standards about selectivity index and lateralization index will also affect AVS results.^34^ Besides, for some patients with severe or refractory hypertension, AVS may be performed without withdrawn drug interference, thereby reducing the accuracy of diagnosis.^35^ Moreover, even if AVS diagnosed patients with unilateral hypersecretion, its internal differences can also affect the outcome of the operation. For example, if AVS shows suppression of the contralateral adrenal gland secretion, there will be a higher possibility of cure of the hypertension^36^ and develop to hyperkalemia^37^ after unilateral adrenalectomy. An adrenal nodule on preoperative imaging referred to improved blood pressure control and preserved renal function after adrenalectomy guided by AVS.^38^

On the other hand, the control of blood pressure is affected by many factors, so the corresponding benefit may not be seen. Complete biochemical success demonstrates a correct diagnosis of unilateral primary aldosteronism and total resection of the pathological adrenal gland.^19^ Although high BMI can increase plasma aldosterone levels because of the positive feedback relationship between the secretion of adipokines leptin and aldosterone,^39^ the biochemical result may still be the best indicator to show the value of AVS. The lack of clinical remission may attribute to not only inappropriate distinguish but also a complication with essential hypertension or chronic kidney disease or subclinical hypercortisolism,^40^ long duration of hypertension, as well as male sex, elderly patients, KCNJ5 mutation carriers,^41^ etc. Proye and coworkers reported that the prevalence of hypertension was almost the same in postoperative patients as the prevalence of essential hypertension in a random population of the same age.^42^ Moreover, the PASO study also reported that the median time for the duration of hypertension was 5 years in patients with complete clinical success after surgery, while 10 years in those with partial or absent clinical success.^19^ Nevertheless, regardless of whether blood pressure and antihypertension drugs improvement, correction of excessive aldosterone and increasing renin concentration themselves reduced risk for cardiovascular events, mortality,^43^ and renal damage,^44^ by inhibiting mineralocorticoid receptor activation. Current studies also suggested that partial or total adrenalectomy may have a different impact on the prognosis of primary aldosteronism.^45^, ^46^ Among included studies, only two studies have reported whether unilateral adrenalectomy was partial or complete^13^, ^15^ and one patient of partial adrenalectomy was performed at the patient's request,^13^ therefore needing further studies to explore the impact of surgical procedure on the prognosis of primary aldosteronism.

As an imaging examination method, CT is difficult to find microadenomas owing to slice thickness and resolution limitation, leading to missing patients who could benefit from surgery. The difference in the left and right adrenal glands' physiological volume^47^ may result in inaccurate interpretation. Meanwhile, the largest adenoma on CT is not always the primary source of aldosterone overdose, and sometimes aldosterone is not produced at all.^48^, ^49^ Some studies have reported larger adrenal tumors or a higher proportion of patients with unilateral adrenal abnormalities in CT‐guided than AVS‐guided patients at baseline, which may make CT‐guided patients more likely to achieve clinical success, suggesting that more clinical trials are needed to confirm the findings of this meta‐analysis. Nonetheless, the existence of CT is indispensable. It can identify aldosterone carcinoma^50^ and guide adrenal vein intubation's success by clarifying the anatomy.^9^

Compared with remission rates obtained in the PASO study, patients guided by AVS had little difference in all success rates except that the proportion of absent clinical success in the PASO study was 15.9%,^19^ but only 9.6% in this systematic review. Possible reasons accounting for this difference. On the one hand, there were different understandings about 0.5 defined daily dose related to definition of absent clinical success provided by PASO consensus,^19^ one was a 50% change,^51^ the other was absolute value of ≥0.5.^52^ On the other hand, most of the included studies did not report the definition of changes in blood pressure and medications, which may lead to underestimation of absent clinical success.

There were some limitations of this study. Firstly, our study lacked hard endpoints, such as the incidence of cardiovascular events and mortality. In the present meta‐analysis, AVS was beneficial to biochemical success compared with CT, but there was no apparent advantage to clinical success. Therefore, further research of the evaluation of hard endpoints is needed. Besides, only one randomized controlled trial was included. After excluding the randomized controlled trial, there was no effect on the overall results, but more randomized controlled trials are still needed to provide stronger evidence.

In conclusions, AVS classification can promote the postoperative biochemical improvement of patients with primary aldosteronism, but it has not been proved beneficial to the advancement of clinical conditions, suggesting the necessity to focus not only on clinical outcomes but also biochemical outcomes when evaluating the prognosis of adrenalectomy in patients with primary aldosteronism. Meanwhile, this study will be meaningful for the popularity of AVS in the future. To obtain a more reliable conclusion in the future, researchers need a stricter diagnosis and treatment process for the primary aldosteronism, standard AVS operation, uniform CT parameters, strict follow‐up, and classification results.

## CONFLICT OF INTEREST

The authors declared no conflict of interest.

## FUNDING

This work was supported by the National Key Research and Development Program of China (2016YFC0900902); Beijing Natural Science Foundation (7212006); and National Natural Science Foundation of China (82073635).

## AUTHOR CONTRIBUTIONS

Y.‐Y. and Y.‐Q. are responsible for the conception and design of the systemic review and meta‐analysis. Y.‐Y. and H.W.‐S. did literature search, reviewed the retrieved items for eligibility, and evaluated the quality of eligible studies. Y.‐Q. was involved in the discussion if there was any disagreement on the eligibility and quality of the studies. Y.‐Y. prepared the first draft of the manuscript. All authors have access to all data, read the manuscript, critically commented and revised the manuscript and gave the final approval.

## Supporting information

Supporting information.
