# Supplementary material for: Prognosis of adrenalectomy guided by computed tomography versus adrenal vein sampling in patients with primary aldosteronism: A systematic review and meta‐analysis
Source: J Clin Hypertens (Greenwich). 2022 Jan 22;24(2):106–15. doi: 10.1111/jch.14395 (PMC8845452; doi:10.1111/jch.14395)
Supplement: Supplementary file 1 — Supporting information. [file JCH-24-106-s001.DOCX]

**ONLINE SUPPLEMENT**

Text S1 Search strategy

Table S1 Diagnosis and classification criteria of primary aldosteronism

Table S2 Quality assessment

Table S3 Definition of clinical success and biochemical success

Table S4 Method for performance of AVS guided by CT image findings

Table S5 Supplement characteristics of the studies meeting the inclusion criteria

Table S6 Surgery outcomes of plasma renin activity and aldosterone-to-renin ratio

Figure S1 Continuous outcome index with CT vs. AVS guided

Figure S2 Supplement clinical and biochemical success rate with CT vs. AVS guided

Figure S3 Sensitivity analyses

Figure S4 Funnel plots

Text S1 Search strategy

a. Search strategy for Pubmed

#1. (hyperaldosteronism[MeSH Major Topic]) OR (primary aldosteronism[Title/Abstract]) OR (hyperaldosteronism[Title/Abstract]) OR (Conn Syndrome[Title/Abstract])

#2. (surgery[Title/Abstract]) OR (adrenalectomy[Title/Abstract])

#3. #1 AND #2

b. Search strategy for Embase

#1. 'hyperaldosteronism'/exp OR 'primary aldosteronism':ab,ti OR 'conn syndrome':ab,ti OR 'hyperaldosteronism':ab,ti

#2. 'surgery':ab,ti OR 'adrenalectomy':ab,ti

#3. #1 AND #2

c. Search strategy for Cochrane library

#1. MeSH descriptor: [hyperaldosteronism] explode all trees

#2. (hyperaldosteronism):ti,ab,kw OR (primary aldosteronism):ti,ab,kw OR (Conn Syndrome):ti,ab,kw

#3. #1 OR #2

#4. (surgery):ti,ab,kw OR (adrenalectomy):ti,ab,kw

#5. #3 AND #4

Table S1 Diagnosis and classification criteria of primary aldosteronism

|  | Diagnosis of primary aldosteronism | Diagnosis of hypokalemia | Diagnosis of hypertension | Standard of distinguishing subtypes by AVS | Slice thickness of CT |
| --- | --- | --- | --- | --- | --- |
| Dekkers et al (2016) | Confirmed by an oral or intravenous salt-loading test | <3.5 mmol/L | ≥135/85 mm Hg using a semiautomatic device, or ≥ 140/90 mm Hg using oﬃce measurement of BP. | ACTH used  SI ≥3  LI ≥4 and suppression index ≤1 | 1 mm |
| Ma et al (2020) | According to the US Endocrine Society guideline, based on an elevated ARR, a confirmatory test of saline infusion test or captopril challenge test is followed. | NA | NA | SI >3 (ACTH used)  SI >2 (no ACTH)  LI ≥4 (ACTH used)  LI ≥2 (no ACTH)  If 3 <LI <4 (ACTH used), determined by clinical manifestations | NA |
| Nwariaku et al (2006) | Confirmed by ARR screening test ratios  saline suppression of 24- hour urinary aldosterone levels (14.0 μg/24 h [39 nmol/d]) after saline suppression. | NA | NA | ACTH used  SI ≥3  LI ≥4 | 2 mm |
| Pirvu et al (2014) | PAC >180 ng/L in the supine  ARR >23 | NA | ≥140/90 mm Hg | No ACTH  SI ≥3  LI ≥3 | NA |
| Rossi et al (2019) | NA | NA | ≥140/90 mm Hg | SI >3 or 5 (ACTH used)  SI >1.36 or 2 or 3 (no ACTH)  LI ≥2 or 3 or 4 or 5 (ACTH used)  LI ≥2 or 3 or 4 or 5 (no ACTH) | NA |
| Tan et al (2006) | PAC ≥15 ng/L  PRA <1 ng/mL/h  ARR >30  OR  PAC ≥15 ng/L  30 >ARR >20  fludrocortisone test  saline suppression test  oral salt loading test | NA | NA | No ACTH | NA |
| Thiesmeyer et al (2020) | PAC ≥15 ng/dL  PRA <1 ng/mL/h  ARR >20 (ng/dL)/(ng/mL/h) | <3.6 mEq/L | ≥140/90 mm Hg | NA | 2.5 mm |
| Williams et al (2018) | Guided by the US Endocrine Society guideline or the Japan Endocrine Society guideline | <3.6 mmol/L | ≥140/90 mm Hg | NA | <3 mm |
| Yeung et al (2020) | An elevated aldosterone level in the setting of suppressed renin and concomitant hypokalemia. Routine confirmatory testing was not obtained. | <3.6 mmol/L | ≥140/90 mm Hg | ACTH used  SI ≥5  LI ≥4 | NA |
| Zarnegar et al (2008) | PAC ≥15 ng/dL  PRA ≤1 ng/mL/h  ARR ≥20 (ng/dL)/(ng/mL/h) | NA | NA | No ACTH  LI ≥4 or 5 | 1.25–3.0 mm |
| Zhu et al (2016) | ARR >240 (ng/dL)/(ng/mL/h) with 2 independent samples and confirmed by either a fludrocortisone  test or a saline infusion test. | NA | ≥140/90 mm Hg | No ACTH  SI ≥3  LI ≥2 | 1.25–3.75 mm |

ARR, aldosterone-to-renin ratio; DRC, direct renin concentration; LI, lateralization index; NA, not available; PAC, plasma aldosterone concentration; PRA, plasma renin activity; SI, selectivity index, defined as the ratio of adrenal venous plasma cortisol concentration to peripheral plasma cortisol concentration, means catheterization successful; Suppression index: the ratio of aldosterone normalised to cortisol between the non-dominant adrenal gland and peripheral blood; LI, lateralization index=(aldosterone/cortisol) _dominant adrenal vein_ / (aldosterone/cortisol) _contralateral adrenal vein_, means lateralization.

Table S2 Quality assessment

A. Cohort studies

| Study | Newcastle-Ottawa Scale | | | | | | | | | | |
| --- | --- | --- | --- | --- | --- | --- | --- | --- | --- | --- | --- |
|  | Selection | | | | Comparability | | | Outcome | | | Total |
|  | 1^a^ | 2^b^ | 3^c^ | 4^d^ | | 5^e^ | 6^f^ | | 7^g^ | 8^h^ |  |
| Ma et al (2020) | 1 | 1 | 1 | 1 | | 1 | 0 | | 1 | 0 | 6 |
| Nwariaku et al (2006) | 1 | 1 | 1 | 1 | | 0 | 1 | | 0 | 0 | 5 |
| Pirvu et al (2014) | 0 | 1 | 1 | 1 | | 0 | 1 | | 1 | 1 | 6 |
| Rossi et al (2019) | 1 | 1 | 1 | 1 | | 2 | 1 | | 1 | 0 | 8 |
| Tan et al (2006) | 0 | 1 | 1 | 1 | | 0 | 1 | | 0 | 1 | 5 |
| Thiesmeyer et al (2020) | 1 | 1 | 1 | 1 | | 1 | 1 | | 0 | 0 | 6 |
| Williams et al (2018) | 1 | 1 | 1 | 1 | | 0 | 1 | | 1 | 1 | 7 |
| Yeung et al (2020) | 0 | 1 | 1 | 1 | | 2 | 1 | | 1 | 1 | 8 |
| Zarnegar et al (2008) | 0 | 1 | 1 | 1 | | 2 | 1 | | 1 | 1 | 8 |
| Zhu et al (2016) | 0 | 1 | 1 | 1 | | 2 | 1 | | 1 | 0 | 7 |

a, representativeness of the exposed cohort; b, selection of the non-exposed cohort; c, ascertainment of exposure; d, demonstration that outcome of interest was not present at start of study; e, comparability of cohorts on the basis of the design or analysis; f, assessment of outcome; g, was follow-up long enough for outcomes to occur; h, adequacy of follow-up of cohorts. When the selective grouping, the average or median follow-up time is less than six months, or the follow-up rate is less than 90%, corresponding to the representativeness of the exposed cohort, follow-up long enough for outcomes, or adequacy of follow-up gets 0 points. When adding up the scores, and the total mark is 9 points, 8-9 points awarded for low risk of bias, 6-7 points refer to moderate risk of bias, 1-5 points apply to high risk of bias, and 0 means a very high risk of bias.

B. Randomized controlled trial

| Source of bias | Item | Risk |
| --- | --- | --- |
| Selection bias | Random sequence generation | low |
|  | Allocation concealment | unclear |
| Performance bias | Blinding of participants and personnel | low |
| Detection bias | Blinding of outcome assessment | low |
| Attrition bias | Incomplete outcome data | low |
| Reporting bias | Selective reporting | low |
| Other bias | Other sources of bias | low |

Only one in the included studies: Dekkers et al (2016).

Table S3 Definition of clinical success and biochemical success

A. Definition of clinical success

|  | Definition of changed antihypertensive | definition of changed BP | Complete success | Partial success | Absent success |
| --- | --- | --- | --- | --- | --- |
| Dekkers et al (2016) | NA | NA | Reached target daytime ambulatory BP without the use of any antihypertensive drugs. Target daytime ambulatory BP was < 135/85 mm Hg. | NA | NA |
| Ma et al (2020) | NA | NA | Normal BP without the aid of antihypertensive medication | Reduced BP or less antihypertensive medication | Unchanged or increased blood pressure with either the same amount or an increase in antihypertensive medication |
| Nwariaku et al (2006) | NA | NA | No antihypertensive medication | NA | NA |
| Pirvu et al (2014) | NA | NA | Normal BP without the aid of antihypertensive medication | Normal BP with a reduced number of antihypertensive  drugs | No change in the number of antihypertensive drugs or uncontrolled hypertension |
| Rossi et al (2019) | >2 drugs | >10% | Normal BP without the aid of antihypertensive medication | Normotension on the same or reduced number of medications, or the same BP as before surgery with less antihypertensive medication, or a reduction in BP with either the same amount or less antihypertensive medication | Unchanged or increased blood pressure with either the same amount or an increase in antihypertensive medication |
| Tan et al (2006) | NA | NA | BP normalized | Less antihypertensive medication | others |
| Thiesmeyer et al (2020) | NA | NA | Normal BP without the aid of antihypertensive medication | The same BP as before surgery with less antihypertensive medication or a reduction in BP with either the same amount or less antihypertensive medication | Unchanged or increased BP with either the same amount or an increase in antihypertensive medication |
| Williams et al (2018) | ≥0.5 DDD | Systolic BP ≥20 mm Hg or diastolic BP ≥10 mm Hg | Normal BP without the aid of antihypertensive medication | The same BP as before surgery with less antihypertensive medication or a reduction in BP with either the same amount or less antihypertensive medication | Unchanged or increased BP with either the same amount or an increase in antihypertensive medication |
| Yeung et al (2020) | ≥0.5 DDD | systolic BP ≥20 mm Hg or diastolic BP ≥10 mm Hg | Normal BP without the aid of antihypertensive medication | The same BP as before surgery with less antihypertensive medication or a reduction in BP with either the same amount or less antihypertensive medication | Unchanged or increased BP with either the same amount or an increase in antihypertensive medication |
| Zarnegar et al (2008) | NA | NA | In addition to partial and absent clinical success | A reduction in the number of medications required for blood pressure control or better control of blood pressure with  the same number of drugs. | Have poor blood pressure control and no reduction in antihypertensive medications |
| Zhu et al (2016) | NA | NA | Normal BP without the aid of antihypertensive medication | Normal BP with a reduced number of antihypertensive  drugs | BP ≥140/90 mm Hg with an increase or no change in the number of preoperative  antihypertensive drugs |

BP, blood pressure; DDD, defined daily dose; NA, not available.

B. Definition of biochemical success

|  | ARR normalized standard | Complete success | Partial success | Absent success |
| --- | --- | --- | --- | --- |
| Dekkers et al (2016) | NA | Resolved aldosteronism | Persistent primary aldosteronism but normokalemia | Hypokalaemia |
| Ma et al (2020) | NA | NA | NA | Hypokalaemia |
| Nwariaku et al (2006) | NA | NA | NA | Hypokalaemia |
| Tan et al (2006) | NA | Aldosterone cure | NA | Hypokalaemia |
| Thiesmeyer et al (2020) | ARR ≤30 | Correction of hypokalaemia and normalization of the ARR | Normokalemia with a raised ARR but with an at least 50% decrease in baseline plasma aldosterone concentration | NA |
| Williams et al (2018) | Defined based on local laboratory reference intervals | Correction of hypokalaemia and normalization of the ARR; in patients with a raised ARR post-surgery, aldosterone secretion should be suppressed in a confirmatory  test | Correction of hypokalaemia and a raised ARR with one or both of the following (compared with pre-surgery): ≥50% decrease in baseline plasma aldosterone concentration; or abnormal but improved post-surgery confirmatory test result | Persistent hypokalaemia or persistent raised ARR, or both, with failure to suppress aldosterone secretion with a post-surgery confirmatory test |
| Yeung et al (2020) | defined on the basis of local laboratory reference intervals | Correction of hypokalaemia and normalization of the ARR; in patients with a raised ARR post-surgery, aldosterone secretion should be suppressed in a confirmatory  test | Correction of hypokalaemia and a raised ARR with one or both of the following (compared with pre-surgery): ≥50% decrease in baseline plasma aldosterone concentration; or abnormal but improved post-surgery confirmatory test result | Persistent hypokalaemia or persistent raised ARR, or both, with failure to suppress aldosterone secretion with a post-surgery confirmatory test |
| Zhu et al (2016) | NA | NA | NA | Persistent hypokalaemia |

ARR, aldosterone-to-renin ratio; NA, not available.

Table S4 Method for performance of AVS guided by CT image findings

| Study | Methods and measurements |
| --- | --- |
| Pirvu et al (2014) | Performed AVS when bilateral lesions were present in the CT image and when a single nodular image was not evident on one of the glands. |
| Tan et al (2006) | When CT find a solitary hypodense adrenal mass ≥ 1 cm with a normal contralateral gland, Tan et al. did not consider AVS on these patients. |
| Yeung et al (2020) | The reasons for not doing AVS included CT showing normal. |
| Zarnegar et al (2008) | Patients were selected to undergo adrenal venous sampling for lateralization of the abnormal adrenal gland if 1) the tumor was smaller than 1.0 cm on CT; 2) the contralateral adrenal gland was enlarged or equivocal; 3) by patient request. |
| Zhu et al (2016) | The CT guidance group included patients whose AVS showed bilateral lesions but who ultimately underwent adrenalectomy. |

Table S5 Supplement characteristics of the studies meeting the inclusion criteria

|  | Age  (years) | Male  (%) | BMI  (kg/m^2^) | SBP  (mm Hg) | DBP  (mm Hg) | Potassium (mmol/l) |
| --- | --- | --- | --- | --- | --- | --- |
| Ma et al (2020) | 47.9 vs 47.7 | 37.9 vs 60 | NA | 152.3 vs 153.6 | 92.4 vs 96.7 | 3.1 vs 2.8 |
| Rossi et al (2019) | 50.9 vs 49.5 | 55.6 vs 61.3 | 28.7 vs 28.4 | 155 vs 154 | 94 vs 93 | 3.5 vs 3.5 |
| Thiesmeyer et al (2020) | 49.9 vs 50.7 | 56.2 vs 60 | NA | 150 vs 143 | 90 vs 87 | 3.4 vs 3.3 |
| Williams et al (2018) | 49.3 vs 50.9 | 43.8 vs 53.4 | 27.2 vs 27.1 | 159 vs 152 | 99 vs 93 | 3.1 vs 3.2 |
| Zarnegar et al (2007) | 52.4 vs 53.6 | 30 vs 51 | NA | 167 vs 170 | 95.5 vs 99.7 | 3.03 vs 3.13 |
| Zhu et al (2016) | 44.2 vs 47.1 | 43.3 vs 53.1 | NA | 142 vs 145 | 89 vs 88 | 3.2 vs 3.3 |
|  | | | | | | |
|  | PAC | | PRA | | ARR | |
| Ma et al (2020) | 270 vs 275 (ng/l) | | 2.1 vs 1.9 (mU/l) | | 98 vs 153 (ng/mU) | |
| Rossi et al (2019) | 28.2 vs 26.8 (ng/dL) | | 0.31 vs 0.25 (ng/mL/h) | | 85.6 vs 83 (ng/dL)/(ng/mL/h) | |
| Thiesmeyer et al (2020) | 30.1 vs 41.0 (ng/dL) | | NA | | 130.9 vs 93.2 | |
| Williams et al (2018) | 923.7 vs 876.6 (pmol/L) | | 2.6 vs 2.6 (pmol/L per minute) | | 419.4 vs 363.3 (pmol/L)/(pmol/L per minute) | |
| Zarnegar et al (2007) | 54.4 vs 39.9 (ng/dL) | | 0.25 vs 0.3 (ng/mL/h) | | 172 vs 173 (ng/dL)/(ng/mL/h) | |
| Zhu et al (2016) | 261 vs 284 (pg/mL) | | 0.26 vs 0.37 (ng/mL/h) | | 1035 vs 737 (pg/mL)/(ng/mL/h) | |

Among the 11 included articles, 6 articles respectively reported baseline characteristics, and the other 5 did not report. Above is CT vs AVS.

ARR, aldosterone-to-renin ratio; BMI, body mass index; DBP, diastolic blood pressure; NA, not available; PAC, plasma aldosterone concentration; PRA, plasma renin activity; SBP, systolic blood pressure.

Table S6 Surgery outcomes of plasma renin activity and aldosterone-to-renin ratio

A. Plasma renin activity

|  | AVS | | CT | | P value |
| --- | --- | --- | --- | --- | --- |
|  | Plasma renin activity | Sample size | Plasma renin activity | Sample size |  |
| Dekkers et al (2016) | 2.74(1.68-4.05) (μg/L per h) | 6 | 0.63(0.38-1.94) (μg/L per h) | 10 | 0.06 |
| Thiesmeyer et al (2020) | 0.8(0.3-2.7)(ng/mL/h) | 45 | 0.7(0.3-1.4) (ng/mL/h) | 80 | 0.47 |
| Williams et al (2018) | 19.2(6.9-38.4) (pmol/L per minute) | NA* | 11.7 (5.7–25.6) (pmol/L per minute) | NA* | 0.001 |

*NA: not available (The two groups total 439 patients)

B. Aldosterone-to-renin ratio

|  | AVS | | CT | | P value |
| --- | --- | --- | --- | --- | --- |
|  | Aldosterone-to-renin ratio | Sample size | Aldosterone-to-renin ratio | Sample size |  |
| Williams et al (2018) | 13.2(5.4-31.0)[(pmol/L)/ (pmol/L per minute)] | NA* | 15.1(7.8–45.1) [(pmol/L)/ (pmol/L per minute)] | NA* | 0.021 |

*NA: not available (The two groups total 439 patients)

A


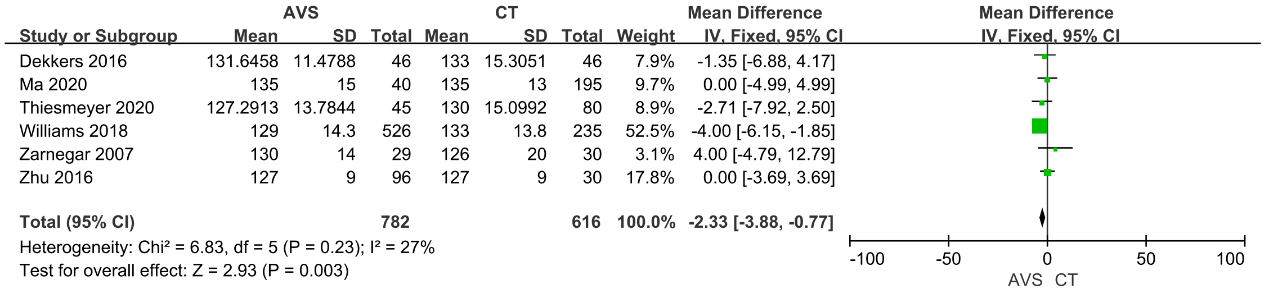


B


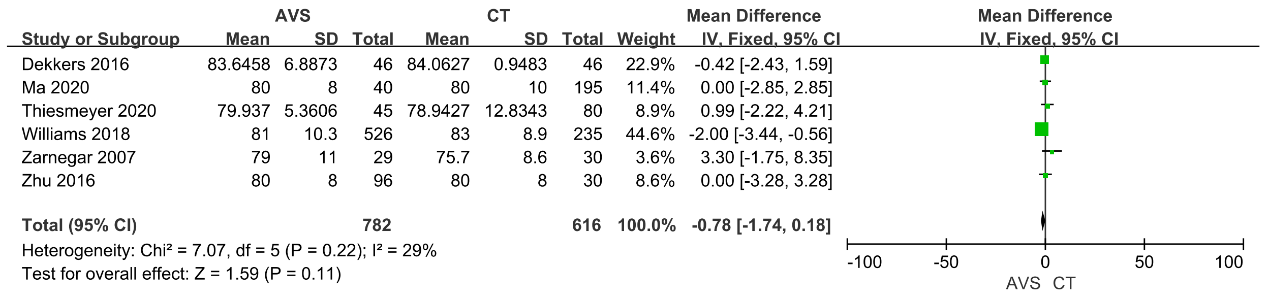


C


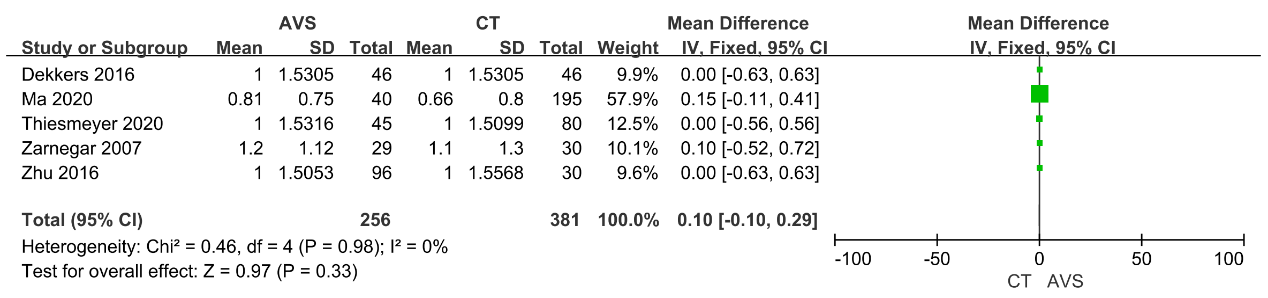


D


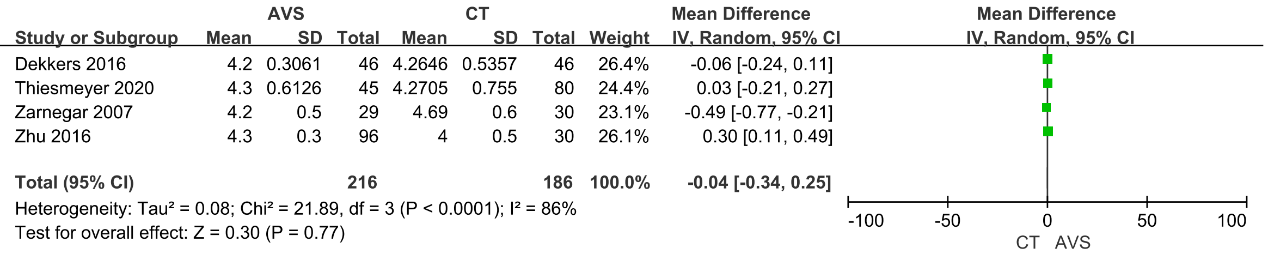


E


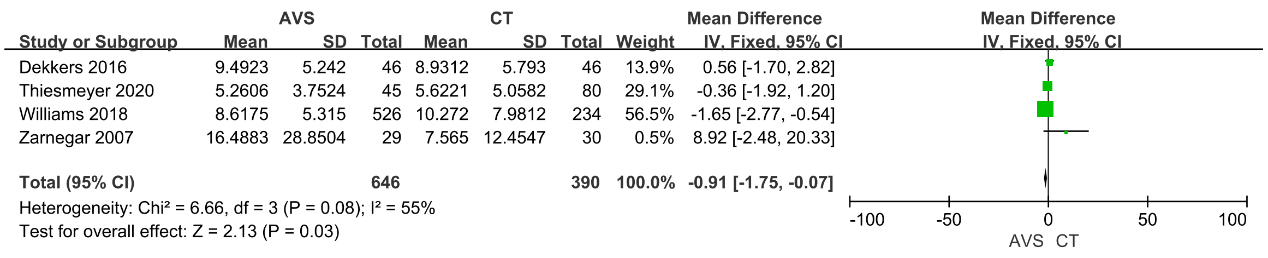


Figure S1 Continuous outcome index with CT vs. AVS guided. The clinical index included systolic blood pressure (A), diastolic blood pressure (B), and the number of antihypertensive drugs (C). The biochemical index had serum potassium (D) and plasma aldosterone concentration (E).

A


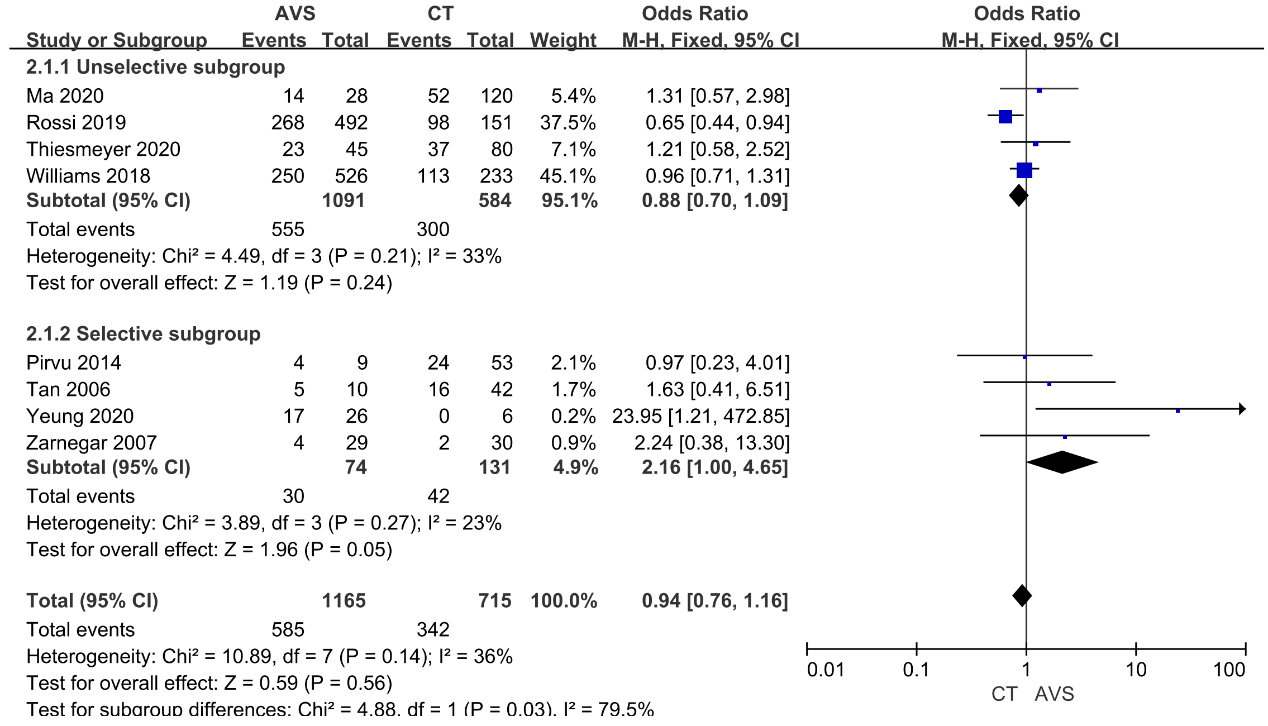


B


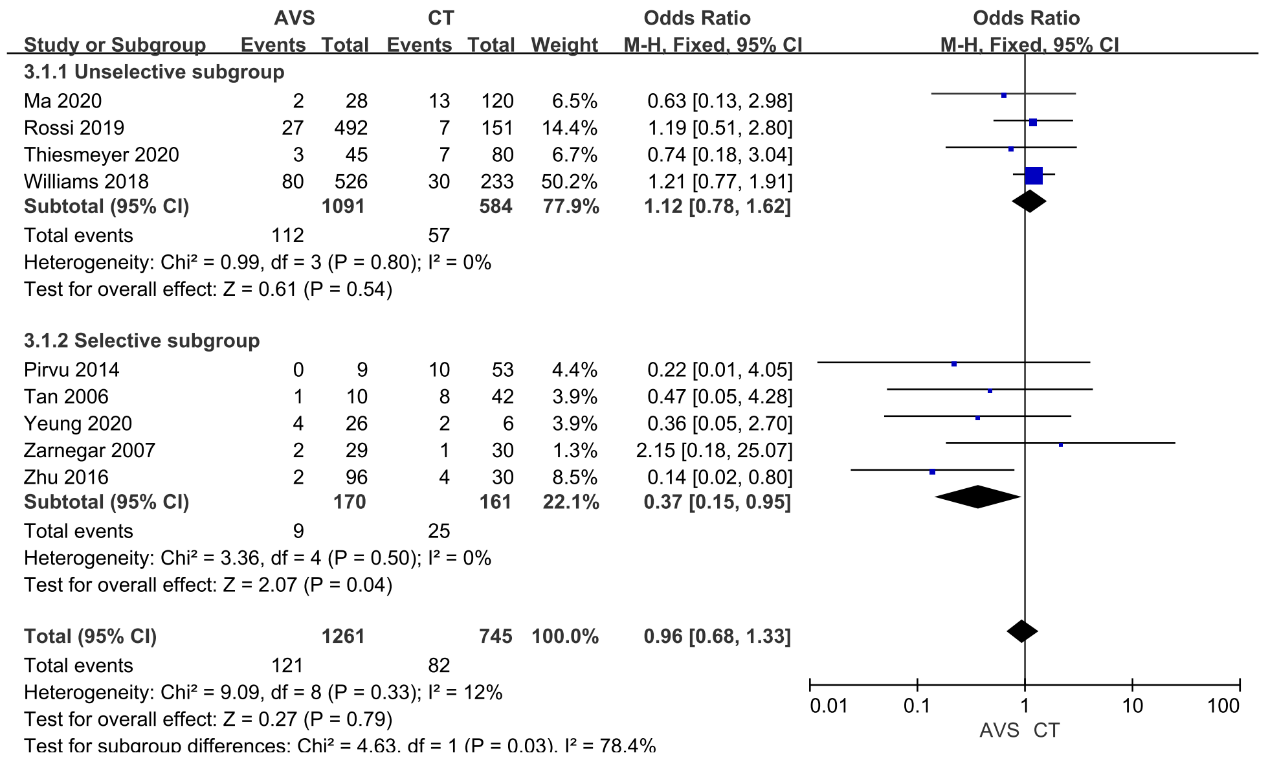


C


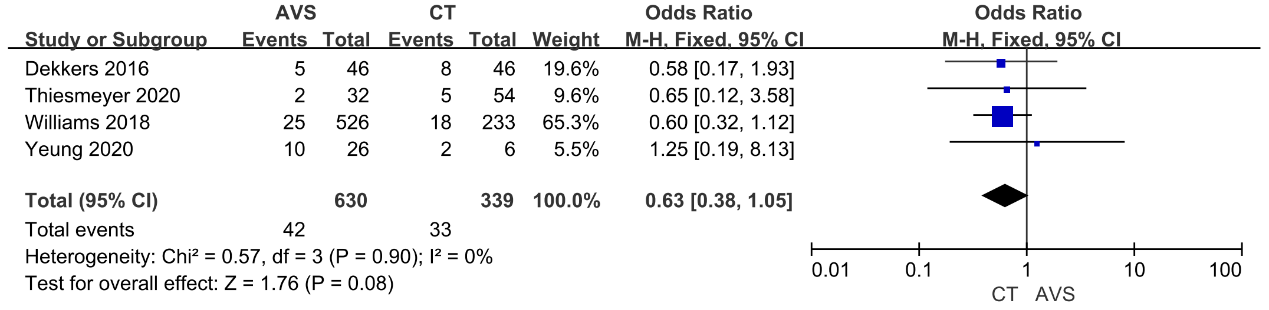


Figure S2 Supplement clinical and biochemical success rate with CT vs. AVS guided. Partial clinical success rate (A), absent clinical success rate (B), and partial biochemical success rate (C).

A


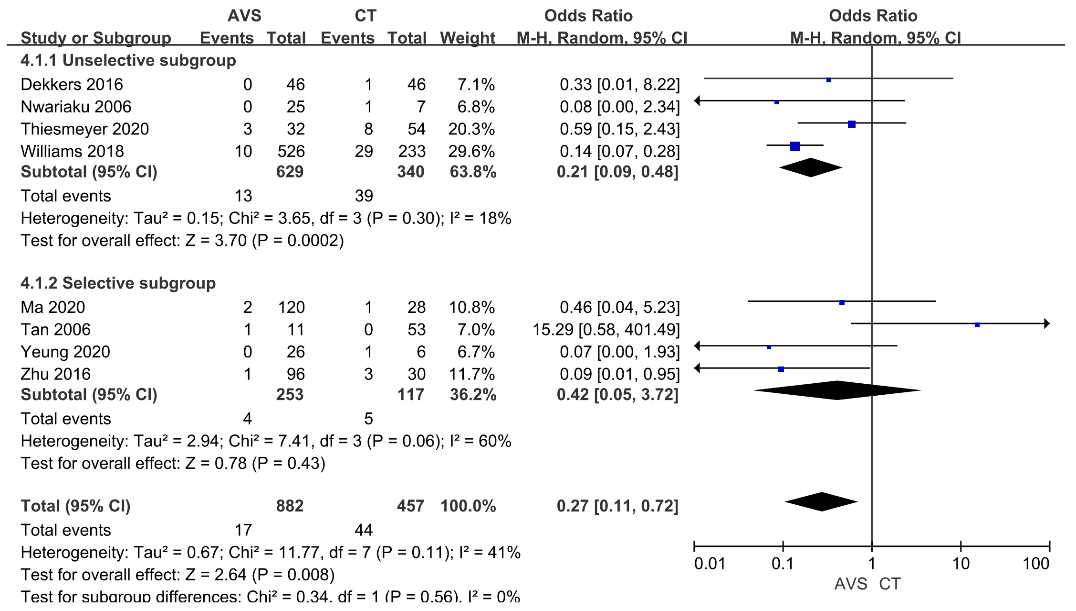


B


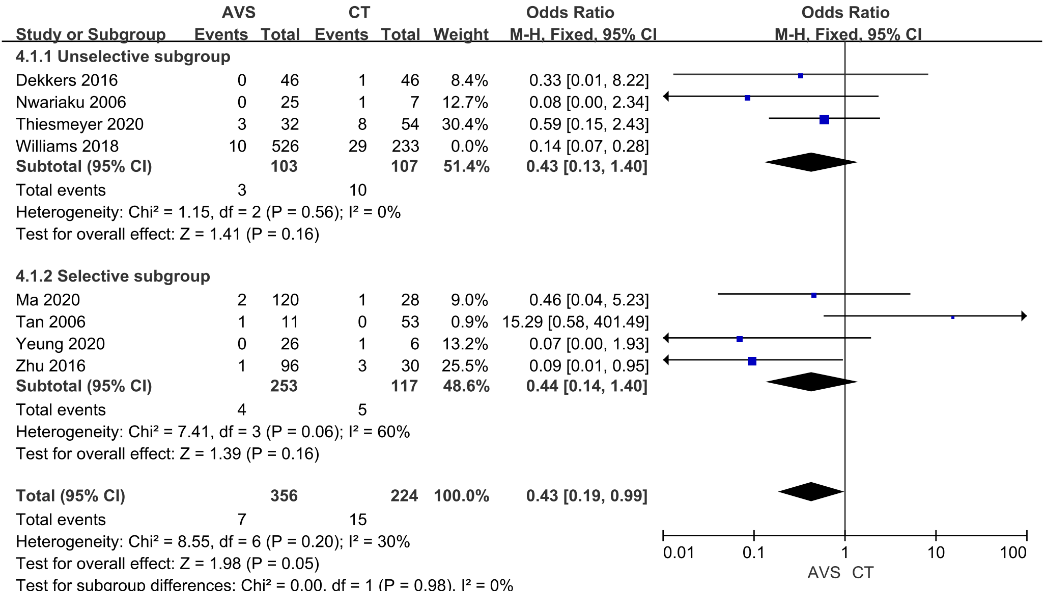


C


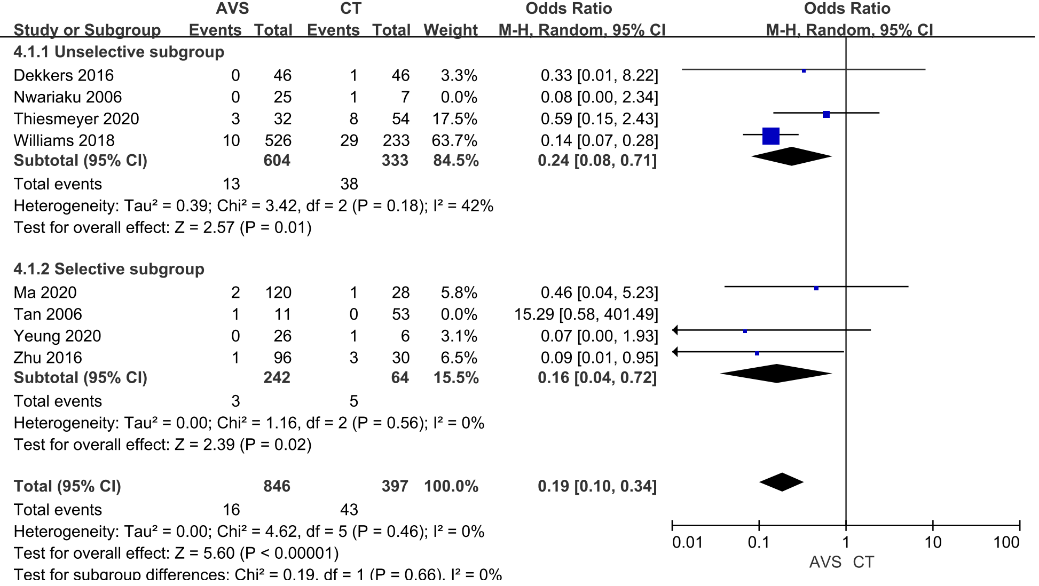


Figure S3 Sensitivity analyses. Absent biochemical success used random-effects model (A) and excluded the largest sample study (B) and high-risk bias studies (C).

A B


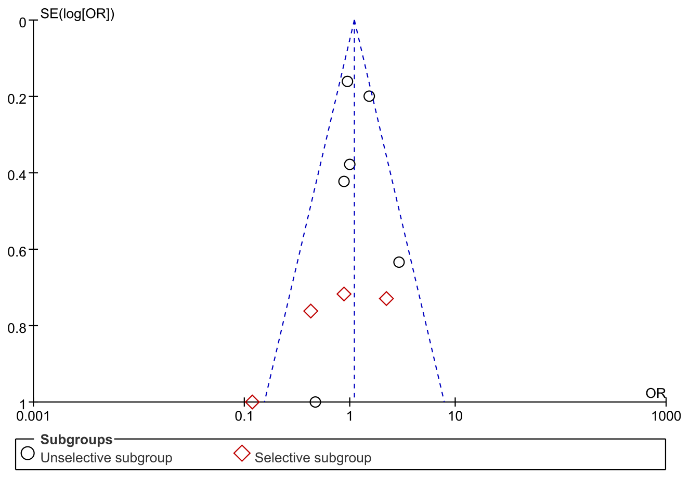

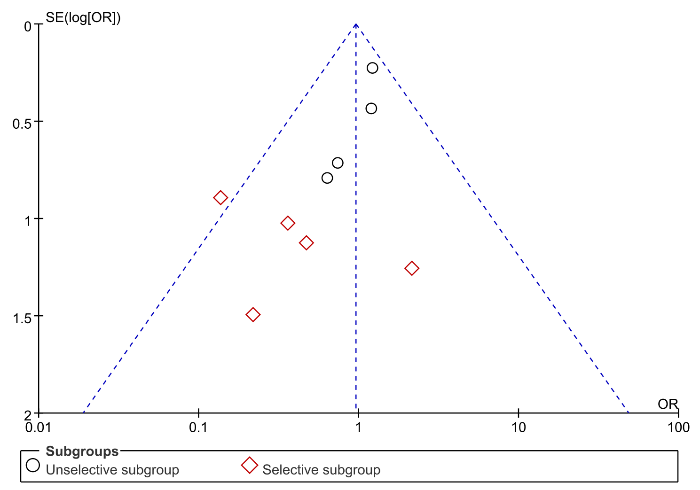


C D


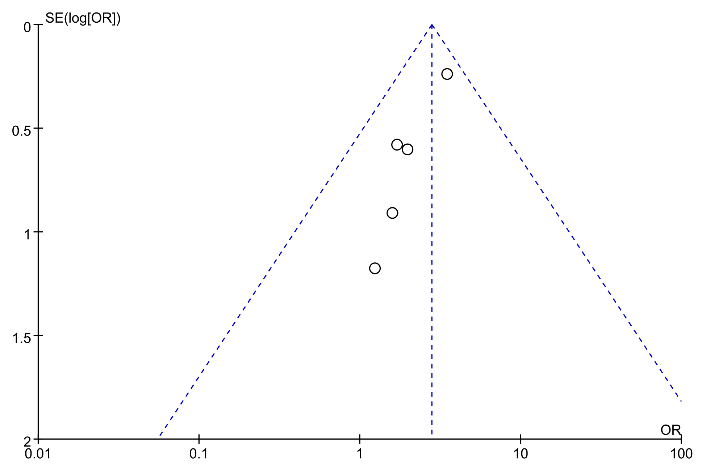

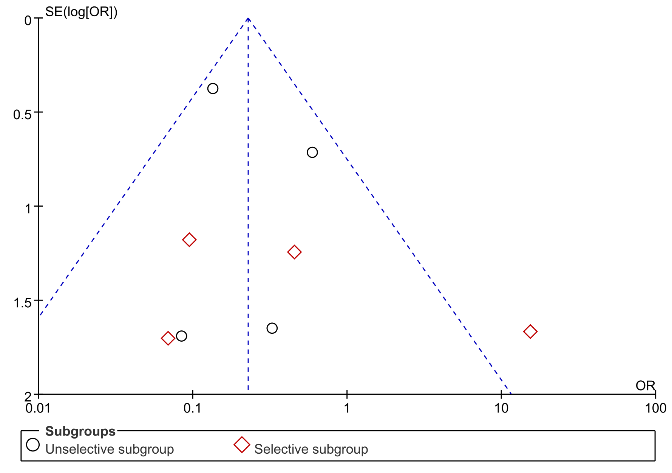


Figure S4 Funnel plots. Complete clinical success rate (A), absent clinical success rate (B), complete biochemical success rate (C), and absent biochemical success rate (D).
